# Learning to Have a Civil Aircraft Take Off under Crosswind Conditions by Reinforcement Learning with Multimodal Data and Preprocessing Data

**DOI:** 10.3390/s21041386

**Published:** 2021-02-16

**Authors:** Feng Liu, Shuling Dai, Yongjia Zhao

**Affiliations:** 1 State Key Laboratory of VR Technology & Systems, Beihang University, Beijing 100191, China; sldai@buaa.edu.cn (S.D.); zhaoyongjia@buaa.edu.cn (Y.Z.); 2 Jiangxi Research Institute, Beihang University, Beijing 100191, China

**Keywords:** autopilot, civil aircraft, multimodal data, reinforcement learning, preprocessing

## Abstract

Autopilot technology in the field of aviation has developed over many years. However, it is difficult for an autopilot system to autonomously operate a civil aircraft under bad weather conditions. In this paper, we present a reinforcement learning (RL) algorithm using multimodal data and preprocessing data to have a civil aircraft take off autonomously under crosswind conditions. The multimodal data include the common flight status and visual information. The preprocessing is a new design that maps some flight data by nonlinear functions based on the general flight dynamics before these data are fed into the RL model. Extensive experiments under different crosswind conditions with a professional flight simulator demonstrate that the proposed method can effectively control a civil aircraft to take off under various crosswind conditions and achieve better performance than trials without visual information or preprocessing data.

## 1. Introduction

The autopilot function of civil aircraft is a technology that allows the aircraft to control itself and complete some flying tasks autonomically, and this technology has been developed over decades. The traditional autopilot function of civil aircraft uses positional data and attitude data usually acquired from sensors in the aircraft to control the plane and guarantee it flies as planned. Examples of tasks accomplished via autopilot include tracking an airline, maintaining smooth flight, maintaining a given airspeed or altitude, and automatically landing by the guidance of an instrument landing system [1,2,3,4].

Although the current autopilot system of civil aircraft has been able to accomplish many flying tasks, it becomes ineffective under severe turbulence or dangerous weather, and at some critical phases, such as taking off and landing, pilots are unwilling to use the autopilot function [5,6,7]. The reason why the traditional autopilot function is not adequate under some emergency or critical conditions is because the traditional autopilot algorithm was designed manually based on aerodynamics, mechanics and control science. However, the abnormal flying conditions are too complex to model manually [8]. As a result, the traditional autopilot function of civil aircraft works only on some simple tasks under calm and common conditions.

The automatic driving of a car based on machine learning has been studied for many years. Some studies use deep learning to process visual data and radar data to improve the cognitive ability of autonomous driving programs on road conditions [9,10]. In order to get a program that can navigate and control a car without using expert data, some studies use reinforcement learning to learn automatically in the environment [11,12]. Civil aviation, as a mode of flying transportation, is very similar to car traffic. Therefore, it is practical and significant to use the most advanced machine learning technology to improve the autopilot capacity of civil aircraft.

A few studies dedicated to the realization of the auto flying of aerial vehicles through advanced machine learning algorithms have been performed in recent years. NASA implemented a project named L2F that used a modified MiG-27 foam target drone and some sensors to conduct real-time aerodynamic modeling and to learn adaptive control [13]. In 2018, Anwar and Raychowdhury successfully made an unmanned aerial vehicle (UAV) learn to fly in a real environment via end-to-end deep reinforcement learning using monocular images [14]. Shaker and Smith presented a fast reinforcement learning algorithm for an unmanned aerial vehicle to learn how to automatically land using visual information [15]. However, most of these works focused on diminutive UAVs and quadcopters, which have simpler structures and are easier to control than complicated and sluggish civil aircraft. In this paper, we focus on a tough task — learning to have a civil aircraft take off under crosswind conditions. Another difference from the previous works is that we use more information to learn to fly. We provide multimodal state data for learning, including real-time flight status data and visual data while the previous works use single-model data; at the same time, we provide preprocessing data that are designed based on general flight dynamics.

Because supervised learning requires a large number of demonstrated samples [16,17] and it is difficult to employ a professional pilot to fly and collect an adequate number of samples, in this study, we use reinforcement learning (RL) [18], which is an unsupervised learning method, to identify the aerodynamics of civil aircraft without a prior aerodynamic knowledge; then, using the RL model to stabilize, control, and navigate the aircraft to accomplish the take-off process. Finally, we perform experiments with a professional flight simulation environment, and the results demonstrate that our machine learning model is able to master this challenging task.

The remainder of the paper is organized as follows—in Section 2, we introduce a few related works. Section 3 introduces the technical background of this paper. In Section 4, we detail the proposed methodology. In Section 5, we describe detailed experimental settings, the results and discussions. In Section 6, we draw a conclusion from our research, discuss a drawback of the proposed method and describe our future studies.

## 2. Related Works

In 2018, Anwar navigated an unmanned aerial vehicle in an indoor real environment via end-to-end reinforcement learning. They used monocular images obtained from the camera in the aircraft as the state information for the RL model. They used double DQN [19] which is a classical RL algorithm to conduct this study and they used the depth of the image to generate the reward. To address safety issues, they created a virtual collision environment to train the aircraft first, and then completed the training in the real environment [14].

In 2019, Koch and Mancuso used reinforcement learning to control the attitude of a quadrotor in an open source high-fidelity simulation environment and utilized digital twinning concepts for minimal effort when transferring trained controllers to hardware [20]. They performed experiments for state-of-the-art RL algorithms on aircraft attitude control, such as deep deterministic policy gradient (DDPG) [21], trust region policy optimization (TRPO) [22] and proximal policy optimization (PPO) [23]. The results show that controllers trained using PPO outperform PID control and are capable of exceptional performance.

In 2020, Xie and Peng presented a reinforcement learning method to make a UAV autonomously track and land on a moving platform. They proposed a partially observable Markov decision process (POMDP). In the POMDP model, the UAV automatically learns the landing maneuver by an end-to-end neural network, which combines the deep deterministic policy gradients algorithm and heuristic rules. They used the position and velocity data of the UAV and the platform as the state information of the RL model, and they used the distance between UAV and moving platform to calculate the immediate reward. The experiments were performed on the Modular Open Robots Simulation Engine. Compared with the PID methods, their method shows good performance when the platform moves in a complex trajectory [24].

Most prior works focused on the small unmanned aerial vehicles and provided single mode data to the RL model. In this study, we try to have a civil aircraft take off autonomously under crosswind conditions by reinforcement learning. Due to the large size and complex mechanical structure of a civil aircraft, we use multi-modal data and preprocessed data to train the learning model. The goal of this work is to improve the ability of the autopilot of civil aircraft under abnormal weather conditions by providing more information and using a more complex RL architecture.

## 3. Technical Background

### 3.1. Reinforcement Learning and DDPG

Reinforcement learning is a branch of machine learning, which learns how to establish policies by exploring an environment without any instructions. RL follows the discounted Markov decision process (S,A,γ,P,r) [25]. Each action $a_{t}\in A$ will lead to a subsequent state $s_{t+1}$ according to the transition probability $P(s_{t=1}|s_{t},a_{t})$ and acquire a reward $r(s_{t},a_{t})$. Using action, state and reward information, the model updates its parameters at each learning step.

The early RL algorithms use a Q-table to record values denoting the learned knowledge and are updated obeying the Behrman equation [26]. Q-learning [27] is a classic RL algorithm that uses a Q-table to record the learned policy. However, since the capacity of the Q-table is limited, if the dimensions of the state or action are excessively high or if a continuous task is being learned, there will be a large amount of transitional value data, and it is difficult for the Q-table to accommodate such a large quantity of data. In recent years, with the development of the artificial neural network (ANN), deep reinforcement learning (DRL), which is a combination of RL and ANN, was proposed [28,29]. DRL possesses both the perception ability of deep learning and the policy-making ability of reinforcement learning, so DRL is competent at performing large-dimension tasks and continuous tasks.

The algorithm proposed in this paper is based on the DDPG method, a popular DRL algorithm that uses an actor-critic structure and outputs deterministic actions rather than a policy distribution. DDPG exhibits outstanding performance on continuous learning tasks and uses 4 networks: an evaluation actor $\mu (s|\theta^{\mu })$, an evaluation critic $Q(s,a|\theta^{Q})$, a target actor $\mu^{\prime }\left(s|\theta^{\mu^{\prime }}\right)$ and a target critic $Q^{\prime }\left(s,a|\theta^{Q^{\prime }}\right)$. The evaluation networks are used to explore and learn, and the target networks are mainly used to give criticism to the evaluation networks. The weights of the target networks are updated by slowly tracking the evaluation networks: $\theta^{\prime }\leftarrow \tau \theta +\left(1-\tau \right)\theta^{\prime }$ with τ≪1. The evaluation critic $Q(s,a|\theta^{Q})$ is optimized by minimizing the loss:

(1) $$L\left(\theta^{Q}\right)=E_{s_{t}∼\rho^{\beta },a_{t}∼\beta ,r_{t}∼E}\left[{\left(Q\left(s_{t},a_{t}|\theta^{Q}\right)-y_{t}\right)}^{2}\right],$$

where

(2) $$y_{t}=r\left(s_{t},a_{t}\right)+\gamma Q^{\prime }\left(s_{t+1},\mu^{\prime }\left(s_{t+1}|\theta^{Q^{\prime }}\right)\right).$$

The evaluation actor $\mu (s|\theta^{\mu })$ is updated by the sampled policy gradient:

(3) $${▽}_{\theta^{\mu }}J\approx \frac{1}{N}\sum_{i}{▽}_{a}Q\left(s,a|\theta^{Q}\right){{|}_{s=s_{i},a=\mu \left(s_{i}\right)}{▽}_{\theta^{\mu }}\mu \left(s|\theta^{\mu }\right)|}_{s_{i}}.$$

### 3.2. Simulation Environment

In this study, we use X-Plane, a professional flight simulator, to conduct the experiments. X-Plane is equipped with functions of advanced flight dynamics simulation, instrument simulation, flight environment simulation and flight operation simulation. X-Plane has been certified by the Federal Aviation Administration of the United States and has been used by the world’s leading defense contractors, air force and aircraft manufacturers for a variety of applications from flight training to conceptual design and flight testing, and also can be used for recording flight experience, private flight license training, instrument flight training. X-Plane is convenient for communicating with external applications by sending flight status data and receiving control commands through the User Datagram Protocol (UDP) or a secondary development plugin.

X-Plane has been used in many studies. A research team of Central Connecticut State University’s School of Engineering have used X-Plane to design a full-scale helicopter simulator [30]. Jirgl and Boril used X-Plane to obtain mathematical identification results of an aircraft model and analyse parameters of mathematical models of human behavior while flying an aircraft [31]. In 2014, Kaviyarasu and Senthil Kumar Simulated a flapping-wing unmanned aerial vehicle using X-plane [32]. Due to the high cost and slow iteration of aircraft design and testing in real environments, X-Plane has been used by many organizations in industry, such as Boeing, NASA, Cessna, Piper, Japan Airlines, and the American Federal Aviation Administration [33].

To communicate with X-Plane, we use X-Plane Connect [34], a plugin developed by NASA, to communicate between X-Plane and our program. The X-Plane Connect (XPC) Toolbox is an open source research tool used to interact with the X-Plane commercial flight simulator and allows users to control an aircraft and receive state information from X-Plane by communicating hundreds of flight data in real time. This research tool has been used to visualize flight paths, test control algorithms, and simulate an active airspace with various aircraft and airlines [35,36].

## 4. Methodology

### 4.1. State Information for Reinforcement Learning

The state information of the environment is referred to as a set of input data for the reinforcement learning model to make a determinative action based on its learned policy, and the components of the input data are critical to the effectiveness of the learning. In this study, the state information consists of 3 sections—(1) The common flight status data; (2) the preprocessing of some flight status data; and (3) visual data from the master pilot’s perspective. Table 1 lists the detailed state information for this study.

#### 4.1.1. Flight Status Data

The flight status of the position, velocity, control, and so forth, can be obtained from sensors in the aircraft or other equipment. Certainly, we cannot obtain these data from the sensors directly; rather, we obtain these data indirectly from the related embedded computer. We also normalize these data to make them more moderate for the neural network. In this study, the flight status we used are as follows:

- **Positional and rotational information:** The positional data include longitude, latitude and altitude, which are denoted as $P_{x}$, $P_{y}$ and $P_{z}$, respectively, in this paper. Generally, this information can be obtained from GPS, ground-based augmentation system or air pressure sensors. The rotational data include the pitch, roll and heading of the aircraft, and these data are denoted as $R_{p}$, $R_{r}$ and $R_{h}$, respectively.
- **Velocity information:** The velocity information of an aircraft includes $V_{x},V_{y},V_{z},V_{p},V_{r}$ and $V_{h}$, which correspond successively to the 3 positional data variables and 3 rotational data variables.
- **True airspeed:** The true airspeed $V_{t}$, which represents the relative speed of the plane and the wind along the heading axis, is also needed and is a critical factor for helping the autopilot system make operational decisions.
- **Wind speed:** The vector of the wind speed $\left(V_{w},O_{w}\right)$ is provided for the RL model, in which $V_{w}$ is the wind speed and $O_{w}$ is the angle between the wind speed and the aircraft heading. In this research, we consider only these two components of the wind speed on the horizontal plane (excluding the wind speed in the vertical direction).
- **Control information:** The control information used in this study is the last control command sent to the aircraft, and it consists of the rudder, elevator, aileron and throttle commands, which are denoted $A_{r},A_{e},A_{a}$ and $A_{t}$, respectively.
- **Deviation from the centerline of the airstrip:** It is necessary to keep the aircraft moving along the centerline of the airstrip during the take-off process, so the deviation from the centerline of the airstrip is input to the autopilot algorithm. To compute the deviation, we establish a coordinate system with the starting point of the airstrip as the origin and transform the position data of the aircraft into this coordinate system. The position of an aircraft in this coordinate system is denoted by the vector P(x,y). G(a,b) is a vector that indicates the direction of the airstrip, and the deviation is defined as
  (4) $$\begin{array}{cc} D & =|\vec{P}|sin<\vec{P},\vec{G}>\\ \; & =\frac{xb-ya}{\sqrt{a^{2}+b^{2}}}. \end{array}$$

#### 4.1.2. Preprocessing Data

Because the aerodynamic model of a civil aircraft is quite complicated and nonlinear and artificial neural networks are not accurate for nonlinear fitting tasks. To improve the nonlinear expression of multilayer networks, we innovatively propose a method that uses preprocessing data as inputs to the RL model. The preprocessing scheme processes some flight status data before they are fed into the model using a trigonometric function, an exponential function, an integral function or any other nonlinear mapping as needed. This method can be regarded as computing parts of mappings of the complex flight dynamics in advance and then using the subsequent neural networks to fit the remaining dynamics.

For a civil aircraft with a conventional wing design, the lift force can be described as

(5) $$Y=\frac{1}{2}C_{y}\rho V^{2}S,$$

where $C_{y}$ is the lift coefficient, ρ is the atmospheric density at the altitude of the aircraft, and *S* is the area of the wing. Under the condition of a mild turbulent flow, *V* is approximately equal to the true airspeed $V_{t}$. Therefore, we define the preprocessing function of the true airspeed as

(6) $$U\left(V_{t}\right)=V_{t}^{2}.$$

**Figure 1:**
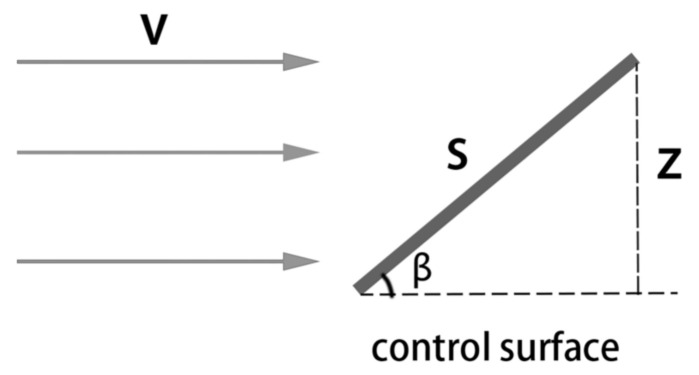
The effective force area for the control surface against the airflow.

Control surfaces such as the rudder, elevator and aileron are mechanisms that control the aircraft’s heading and posture by interacting with the surrounding atmosphere. In general, a rotational command from the flight control computer comprises angular data within a specific range; however, as Figure 1 shows, the force between the control surface and the airflow is proportional not to its rotation angle β but to the effective force area:

(7) Z=S×sin(β),

where *S* is the area of the control surface. Therefore, we define the preprocessing function for the 3 control data variables $\left(A_{r},A_{e},A_{a}\right)$ as

(8) $$U\left(A_{i}\right)=sin\left(A_{i}\right).$$

**Figure 2:**
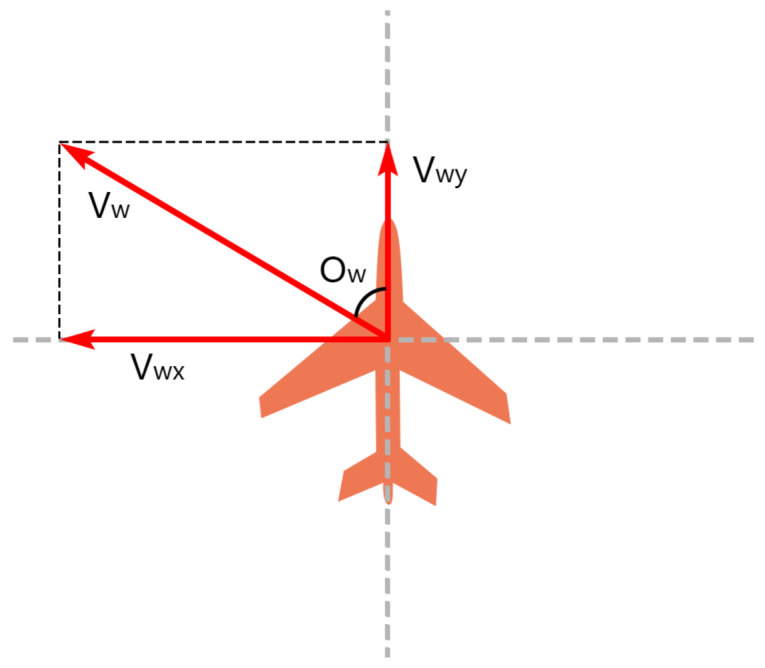
A schematic diagram of wind speed decomposition.

As Figure 2 shows, the wind speed $\left(V_{w},O_{w}\right)$ can be described as a divided, and this symmetric format may be easier for neural networks to understand. Therefore, we provide an additional preprocessed scheme $\left(V_{wx},V_{wy}\right)$ for the wind speed for the learning model:

(9) $$\left\{\begin{array}{c} V_{wx}=V_{w}\times sin\left(O_{w}\right)\\ V_{wy}=V_{w}\times cos\left(O_{w}\right). \end{array}\right.$$

#### 4.1.3. Visual Information

**Figure 3:**
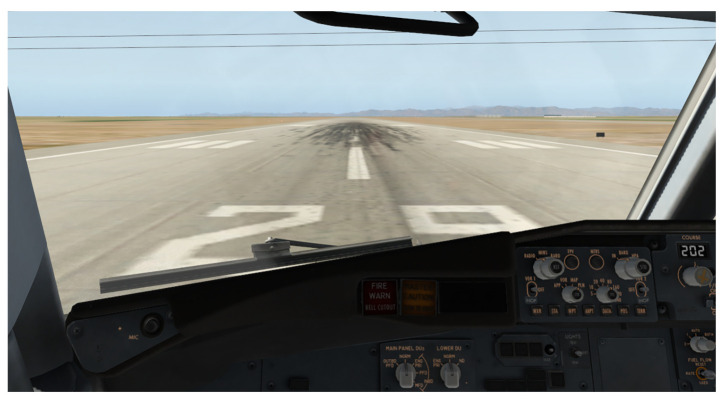
A scene looking out from the main pilot’s position.

A scene obtained by looking out from the main pilot’s position implicitly contains not only the motion information but also the spatial information in front of the aircraft; this information is not available from common sensors. As Figure 3 shows, the visual information we use in this paper is composed of images, namely, screenshots from the video stream of the flight simulator. After a screenshot, we resize the image to be uniform.

### 4.2. Reward Function

In reinforcement learning, a reward function is a regulation that evaluates actions and should be designed according to expert experience. In this paper, to enable the aircraft to move along the airstrip and take off and reach the target area, the reward function is designed to comprise out-of-bounds punishments and rewards for tentative movements.

#### 4.2.1. Out-of-Bounds Punishments

**Figure 4:**
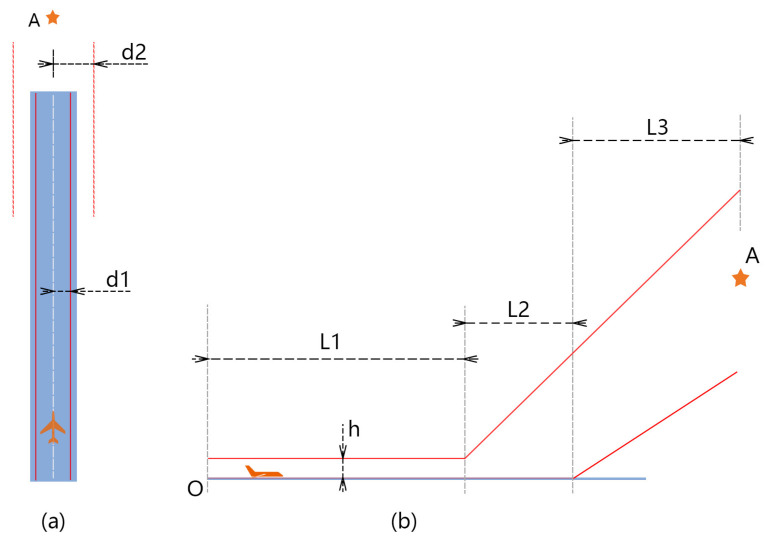
The available area for the aircraft when learning to take off. Panel (**a**) shows the horizontal constraints, and panel (**b**) shows the constraints in the vertical direction.

We set boundaries to constrain the aircraft’s movement within the expected area *B*. In Figure 4, these boundaries are marked with red lines. In the horizontal direction, when the aircraft is on the runway, it should keep moving within the two red lines that are at a distance $d_{1}$ from the centerline, and when the aircraft leaves the ground, it should fly within the bounds of the two red dotted lines at a distance $d_{2}$ from the centerline. In the vertical direction, at stages $L_{1}$ and $L_{2}$, the altitude of the aircraft should not be higher than the red line, and at stage $L_{3}$, the aircraft should fly between the two red lines. If the aircraft flies out of bounds after a tentative action, it will receive a punishment $r_{p}$.

#### 4.2.2. Rewards for Tentative Movements

In the learning process, each tentative movement will obtain an immediate reward that represents the value of the attempt. The goal of the autopilot program is to enable the aircraft to overcome crosswind interference, learn to move on the runway, and learn to take off; thus, the closer the aircraft is to the centerline of the runway, the higher the reward. We define this reward relative to the deviation from the centerline as

(10) $$r_{d}=\partial_{d}\frac{1}{D^{2}},$$

where *D* is defined at Equation 4 and $\partial_{d}$ is a scaling factor.

Target point $A(x_{a},y_{a},z_{a})$ is assigned by the experience of a professional pilot. At step t, the vector $P_{t+1}\left(x_{t+1},y_{t+1},z_{t+1}\right)$ denotes the new position of the aircraft, and Pt(xt,yt,zt) denotes the previous position. $L_{t+1}$ denotes the distance between $P_{t+1}$ and the target point, and $L_{t}$ is the distance between $P_{t}$ and the target point. The difference between $L_{t}$ and $L_{t+1}$ is also used as part of the reward and is defined as

(11) $$r_{l}=\partial_{p}\left(L_{t}-L_{t+1}\right),$$

where

(12) $$L_{t}=\sqrt{{\left(x_{t}-x_{a}\right)}^{2}+{\left(y_{t}-y_{a}\right)}^{2}+{\left(z_{t}-z_{a}\right)}^{2}}$$

and

(13) $$L_{t+1}=\sqrt{{\left(x_{t+1}-x_{a}\right)}^{2}+{\left(y_{t+1}-y_{a}\right)}^{2}+{\left(z_{t+1}-z_{a}\right)}^{2}}.$$

From the above discussion, the reward function is defined as

(14) $$\left\{\begin{array}{cc} r=r_{d}+r_{l}\; & :p_{t+1}\in B\\ r=r_{p}\;\;\; & :p_{t+1}\notin B. \end{array}\right.$$

### 4.3. Experience Replay

The DDPG structure on which our method based is an off-policy reinforcement learning method, and experience replay is an important component for off-policy learning [21]. At step *t*, the transition data that are collected and used to train the model consist of flight status data, visual data, the action, the reward, the next flight status data and the next visual data, and the transition is denoted as $T_{t}=\left[S_{s_{t}},S_{i_{t}},A,R,S_{s_{t+1}},S_{i_{t+1}}\right]$.

**Figure 5:**
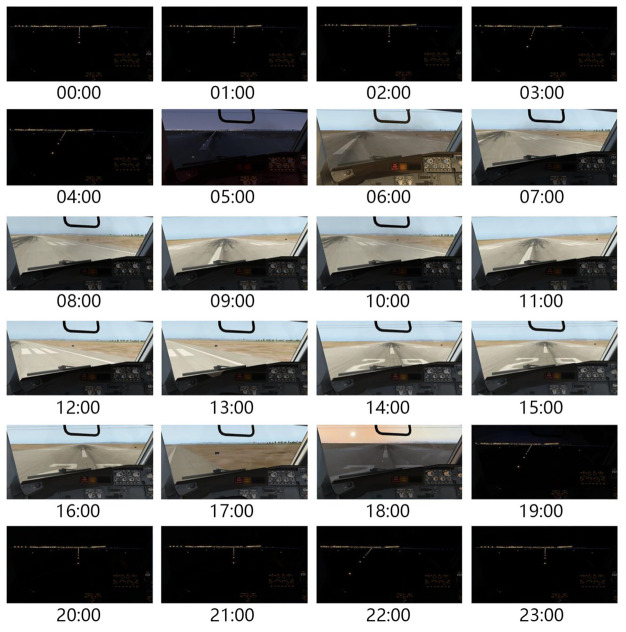
The change in a scene looking out from the aircraft cockpit over 24 h.

As Figure 5 shows, with changes in sunlight, the scene from the cockpit will vary over the course of a day. The diurnal cycle of sunlight is 24 h, and the experience memory of reinforcement learning is hardly capable of storing such a large amount of image data. As a result, new information will overwrite previous images, causing an incomplete data distribution in the experiential memory. To address this issue, we create 24 independent memories, where each memory works only in the corresponding hour. At each transition collection, we use the prioritized experience replay method [37] that store transitions based on priorities involved with temporal difference error (TD-Error) [38], and the new transition is stored in a certain memory corresponding to the current hour. When training the autopilot model, we select an equal number of transitions from each memory to constitute a batch.

### 4.4. Architecture of RL Model

**Figure 6:**
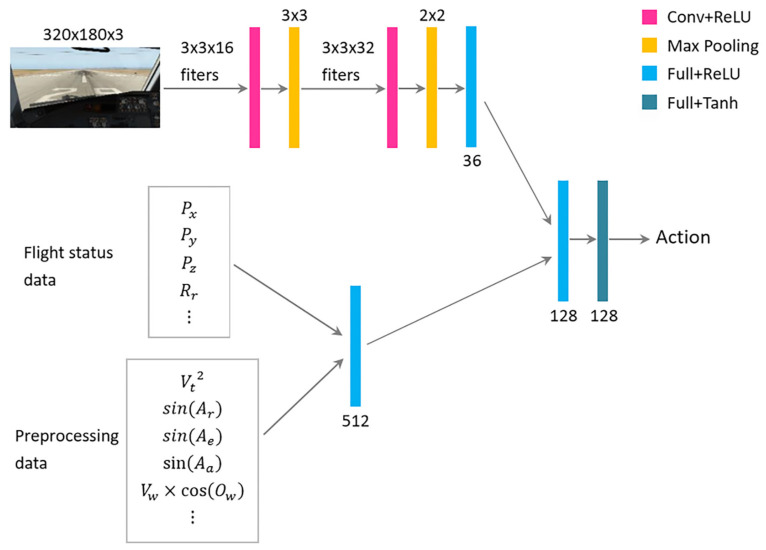
The architecture of the actor network in the proposed reinforcement learning model.

In this study, the reinforcement learning algorithm which is used to learn the autopilot function exhibits an actor-critic structure that based on DDPG. As Figure 6 shows, the actor network accepts multimodal data and outputs the next action $A=(A_{r},A_{e},A_{a},A_{t})$. The fight status data and preprocessing data are fused through a fully-connected layer. The image data, which are resized to 320∗180∗3, are processed by 2 convolutional layers, 2 max-pooling layers and a fully-connected layer. Finally, these feature data are integrated into 2 fully-connected layers.

**Figure 7:**
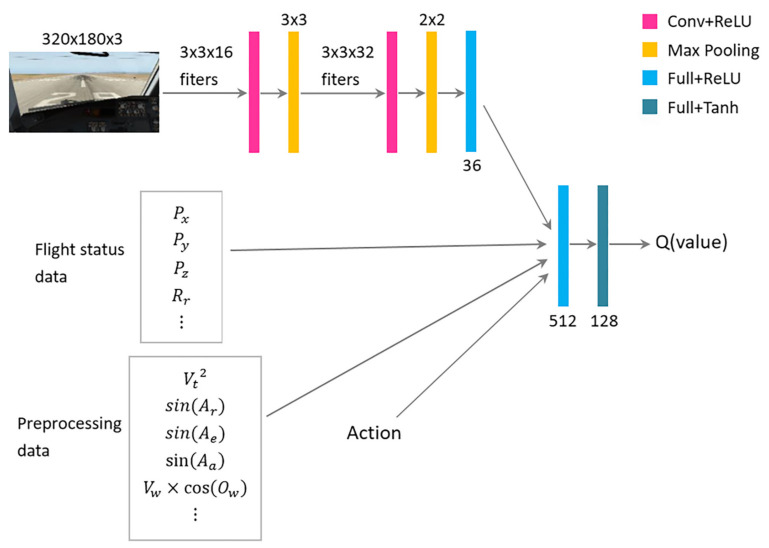
The architecture of the critic network in the proposed reinforcement learning model.

The critic network has a similar structure to the actor network when processing the image data, and additional action data are offered as input information. The number of units in the last two fully-connected layers is different from that within the actor network. Figure 7 illustrates the structure of the critic network in detail.

### 4.5. Implementation Details

In this study, we perform experiments with the flight simulator X-Plane, a Boeing 737 model and the research tool—X-Plane connect. The RL program is compiled in Python and Tensorflow, and each trial has 3000 min learning. The X-Pane simulator and the RL program are run on the same computer, and Table 2 lists the interacting data between them. The visual data are continuously collected by taking screenshots of the X-Plane window. At the start of each episode, the aircraft is reset and placed at the starting point of the runway.

Based on the DDPG algorithm, the actor network and the critic network both have two sets: one is called the evaluation network, and the other is called the target network. The evaluation network containing the current policy is used for learning within the environment and to make action decisions $A_{t}$ and the critic $Q(S_{t},A_{t})$. Because the correlation between $S_{t}$ and $S_{t+1}$ will make the critic $Q(S_{t+1},A_{t+1})$ inaccurate, the target network containing the policies of the previous few steps is used to give the critic $Q^{\prime }\left(S_{t+1},A_{t+1}\right)$. Then, the TD-Error that used in experience replay can be computed by $E_{t}={\left(\left(r+\gamma Q^{\prime }\left(S_{t+1},A_{t+1}\right)\right)-Q\left(S_{t},A_{t}\right)\right)}^{2}$. Table 3 shows the detailed hyperparameter configurations for the proposed RL algorithm. The Algorithm 1 shows the core steps in pseudocode, the input data of this algorithm is the state information $S_{t}$, and the output is the target actor $\mu^{\prime }\left(s|\theta^{\mu^{\prime }}\right)$.
**Algorithm 1** Core steps of the proposed RL algorithm    Randomly initialize eval critic network $Q(s,a|\theta^{Q})$ and eval actor $\mu (s|\theta^{\mu })$ with weights $\theta^{Q}$ and $\theta^{\mu }$.    Initialize target network $Q^{\prime }$ and $\mu^{\prime }$ with weight $\theta^{Q^{\prime }}\leftarrow \theta^{Q}$, $\theta^{\mu^{\prime }}\leftarrow \theta^{\mu }$    Initialize experience memory *M*    Initialize the actor replacement counter $C_{a}=0$    Initialize the critic replacement counter $C_{c}=0$    Initialize the actor replacement interval $I_{a}$    Initialize the critic replacement interval $I_{c}$    **for**
episode=1 to *Z*
**do**        Initialize a random process N for action exploration.        Initialize the aircraft and observe the state $s_{t}$.        **for**
step=1 to *L*
**do**            Select action $a_{t}=\mu \left(s_{t}|\theta^{\mu }\right)+N_{t}$ according to the current policy and exploration noise.            Run action $a_{t}$ and compute reward $r_{t}$ according to the method in Section 3.2.            Observe new state $s_{t+1}$            Store transition $T_{t}=\left(s_{t},a_{t},r_{t},s_{t+1}\right)$ in *M*.            Sample K transitions $\left(s_{i},a_{i},r_{i},s_{i+1}\right)$ from *M*.            Set $y_{i}=r_{i}+\gamma Q^{\prime }\left(s_{i+1},\mu^{\prime }\left(s_{i+1}|\theta^{u^{\prime }}\right)|\theta^{Q^{\prime }}\right)$            Update the critic by minimizing the loss: $L=\frac{1}{N}\sum_{i}{\left(y_{i}-Q\left(s_{i},a_{i}|\theta^{Q}\right)\right)}^{2}$            Update the actor policy using the sampled policy gradient (Eq.3).            **if**
$C_{a}\%I_{a}==0$
**then**
                $\theta^{\mu^{\prime }}\leftarrow \tau \theta^{\mu }+\left(1-\tau \right)\theta^{\mu^{\prime }}$
            
**end if**
            **if**
$C_{c}\%I_{c}==0$
**then**
                $\theta^{Q^{\prime }}\leftarrow \tau \theta^{Q}+\left(1-\tau \right)\theta^{Q^{\prime }}$
            **end if**
            Update $C_{a}$ and $C_{c}$ by $C_{a}=C_{a}+1$; $C_{c}=C_{c}+1$        **end for**
    **end for**
    Get the target actor $\mu^{\prime }\left(s|\theta^{\mu^{\prime }}\right)$.

## 5. Experiments

### 5.1. Experiment 1: Learning to Take Off under Crosswind Conditions at Different Speeds

In this experiment, we conduct 4 trials under crosswind conditions (10 knots at 90${}^{\circ }$, 18 knots at 90${}^{\circ }$, 12 knots at 135${}^{\circ }$, and 12 knots at 45${}^{\circ }$, and we compute the average reward by

(15) $$R_{a}=0.9\times R_{a^{\prime }}+0.1\times \sum_{t=1}^{N}r_{t},$$

where, $R_{a^{\prime }}$ is the last recorded average reward, and *N* is the total number of steps in one episode.

**Figure 8:**
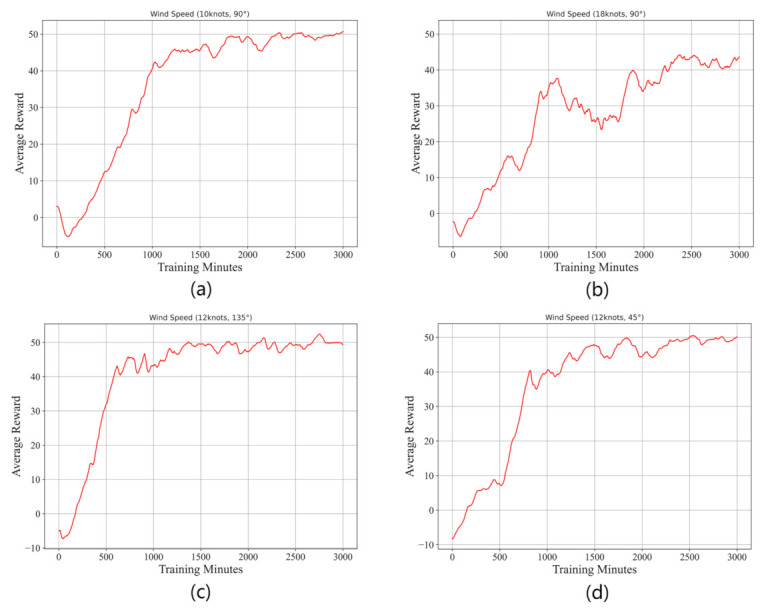
The learning curves of the proposed reinforcement learning algorithm under crosswind conditions at different speeds: (**a**) shows the result of a trial in a 10-knot, 90${}^{\circ }$ crosswind; (**b**) shows the result of a trial in an 18-knot, 90${}^{\circ }$ crosswind; (**c**) shows the result of a trial in a 12-knot, 135${}^{\circ }$ crosswind; and (**d**) shows the result of a trial in a 12-knot, 45${}^{\circ }$ crosswind.

**Figure 9:**
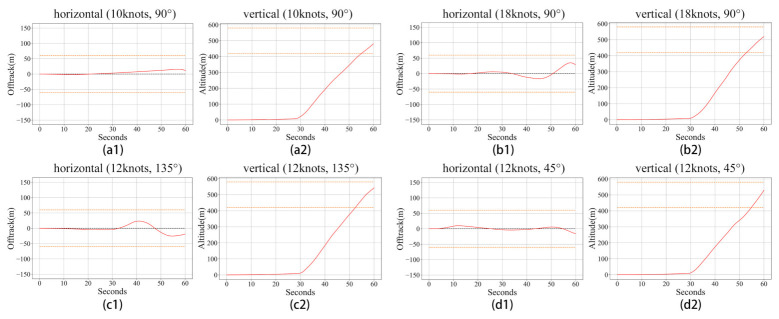
The performance of takeoff under different crosswind conditions after learning. The red line shows the motion trail of the aircraft when it takes off. In each figure, the area between two orange dotted lines is the target area designed based on experience. (**a1**): the motion trail of the horizontal direction in the 10-knot, 90°crosswind; (**a2**): the motion trail of the vertical direction in the 10-knot, 90°crosswind; (**b1**): the motion trail of the horizontal direction in the 18-knot, 90°crosswind; (**b2**): the motion trail of the vertical direction in the 18-knot, 90°crosswind; (**c1**): the motion trail of the horizontal direction in the 12-knot, 135°crosswind; (**c2**): the motion trail of the vertical direction in the 12-knot, 135°crosswind; (**d1**): the motion trail of the horizontal direction in the 12-knot, 45°crosswind; (**d2**): the motion trail of the vertical direction in the 12-knot, 45°crosswind.

**Results and discussion:**Figure 8 shows the learning curves of these trials conducted under crosswind conditions at different speeds. From this figure, we can note that the proposed algorithm can allow a civil aircraft to learn to take off under various crosswinds. In the first 1000 min, the learning performance rise rapidly, and then the learning get into a slow ascent stage accompanied by small shocks. Comparing panel (a) and panel (b), it is obvious that a faster wind speed will make the learning harder and decrease the performance. From panel (c) and panel (d), we can know that the model can learn well both under headwind and tailwind conditions. Figure 9 shows the motion trails of the aircraft when it takes off, and we can find that under different crosswinds, the aircraft can fly within the requested horizontal area and reach the target altitude area at the specified time. Same as the result in Figure 8, the lower the wind speed, the better the flight performance.

### 5.2. Experiment 2: Comparison of Learning with and without Visual Data

In order to observe the implications of visual information, we conduct a trial using our proposed method without supplying visual data. This trial is conducted in a 10-knot, 90${}^{\circ }$ crosswind. The way of computing the average reward is similar to that of Experiment 1.

**Figure 10:**
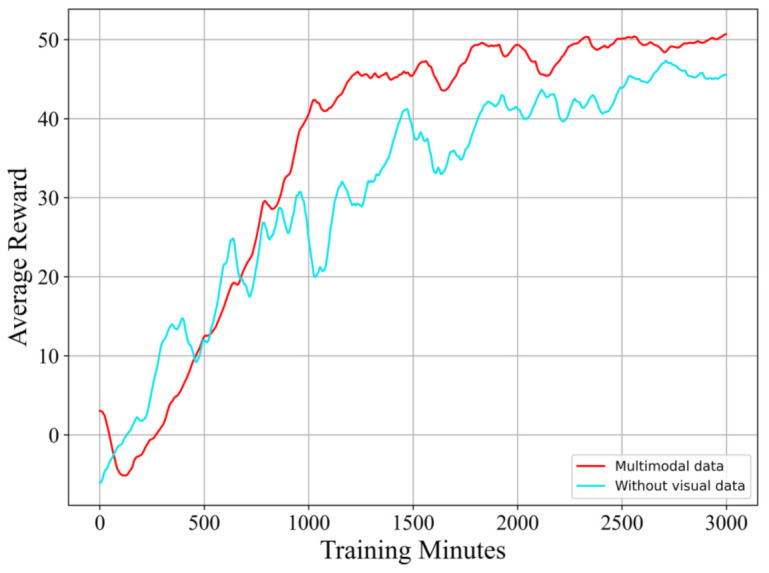
A comparison of the learning curves from the proposed reinforcement learning algorithm with and without visual data.

**Figure 11:**
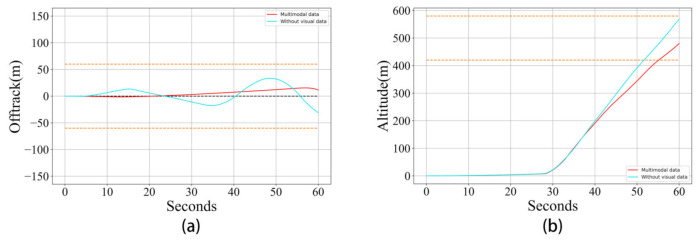
A comparison of the takeoff performance of the proposed reinforcement learning algorithm with and without visual data. (**a**): the compared motion trails of the horizontal direction; (**b**): the compared motion trails of the vertical direction.

**Results and discussion:**Figure 10 clearly shows a comparison of the learning curves from the proposed method with and without visual data. This demonstrates that learning with visual data is more stable and can ultimately acquire a higher score. Though the learning without visual data has a faster learning rate at the initial stage, this advantage fades away after learning for a few hundred minutes. Figure 11 also indicates that the RL model using visual data can make the flight more stable, and the distance to the target point is also closer at the end of the flying.

### 5.3. Experiment 3: Comparison of Learning with and without Preprocessing Data

In this experiment, we conduct a trial using our proposed method without supplying preprocessing data, and compare its learning performance with that of the original proposed method. Similar to Experiment 2, this trial is also conducted in a 10-knot, 90${}^{\circ }$ crosswind, and the same way of computing average reward as that of Experiment 1 is used.

**Figure 12:**
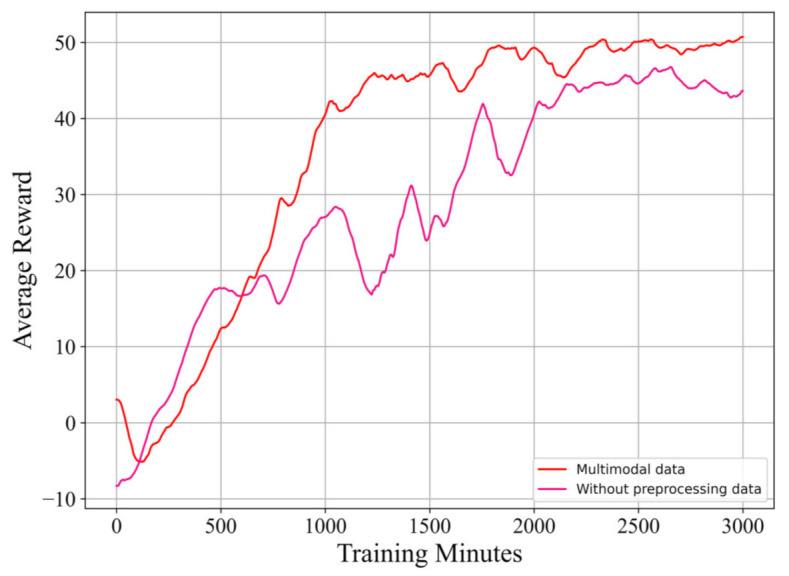
A comparison of the learning curves from the proposed reinforcement learning algorithm with and without preprocessing data.

**Figure 13:**
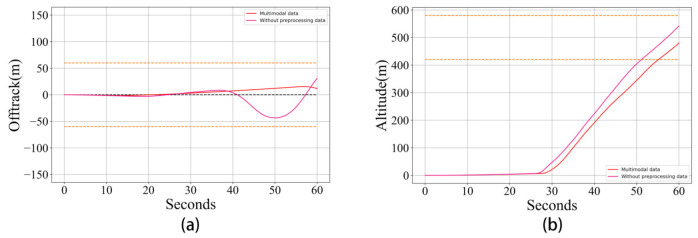
A comparison of the takeoff performance of the proposed reinforcement learning algorithm with and without preprocessing data. (**a**): the compared motion trails of the horizontal direction; (**b**): the compared motion trails of the vertical direction.

**Results and discussion:** As Figure 12 shows, compared with the trial without supplying preprocessing data the trial with preprocessing data can achieve better performance in many aspects, including a better learning stability, faster convergence rate and higher final score. This experiment clearly demonstrates the positive influence brought about by supplying preprocessing data. From Figure 13 we can clearly see that the model using preprocessed data can make the flight trail swing less when taking off.

## 6. Conclusions and Future Studies

In this work, we proposed a reinforcement learning method to learn to accomplish a challenging task—learning to have a civil aircraft take off under crosswind conditions. Our method feeds common flight data, visual data and preprocessing data into the RL model. Experiments under different crosswind conditions demonstrated that the proposed method can effectively accomplish this learning task. Additionally, further comparative experiments indicated the advantages of supplying multimodal data and preprocessing data in the learning. Compared with traditional autopilot algorithms, the proposed algorithm can complete more complex autopilot tasks, and it can be easily applied to other autonomous flying tasks. Using unsupervised machine learning methods, it can reduce a lot of manual modeling work that differ from task to task.

A drawback of our method that is worthy of discussion is that the use of multimodal data and preprocessing data will complicate the architecture of the RL network and use more nerve units. As a result, more GPU memory and more learning hours will be required. However, this issue will become increasingly negligible with the development of computer hardware year after year. The main purpose of this research is to study whether machine learning is capable of the autopilot of aircraft under abnormal weather conditions, however the stability of the RL model cannot be checked, so, considering security, the proposed method can only work on a flight simulator, and it can be used to train pilots in a flight simulation environment. In subsequent studies, we plan to make machine learning and traditional control algorithms work together so that can make full use of the intelligent advantages of machine learning and the stable advantages of traditional control algorithms for improving the ability of autopilot of aircrafts. In addition, we will use machine learning to try to accomplish more difficult autonomous flight tasks, such as implementing the autopilot function under wind shear conditions and learning to have an aircraft recover from stalling.

## Figures and Tables

**Table 1:** The state data fed into the reinforcement learning model.

Symbol	Description
$P_{x}$	Longitude
$P_{y}$	Latitude
$P_{z}$	Altitude
$R_{p}$	Pitch
$R_{r}$	Roll
$R_{h}$	Heading
$V_{x}$	Velocity in the longitude direction
$V_{y}$	Velocity in the latitude direction
$V_{z}$	Velocity in the altitude direction
$V_{p}$	Rotational velocity of pitch
$V_{r}$	Rotational velocity of roll
$V_{h}$	Rotational velocity of heading
$V_{t}$	True airspeed
$V_{w}$	Wind speed
$O_{w}$	The angle between wind speed and the aircraft heading
$A_{r}$	The last control command on the rudder
$A_{e}$	The last control command on the elevator
$A_{a}$	The last control command on the aileron
$A_{t}$	The last control command on the throttle
*D*	Deviation from the centerline of airstrip
$V_{t}^{2}$	Preprocessing function for true airspeed
$sin(A_{r})$	Preprocessing function for rudder control
$sin(A_{e})$	Preprocessing function for elevator control
$sin(A_{a})$	Preprocessing function for aileron control
$V_{w}\times sin\left(O_{w}\right)$	Preprocessing function for wind speed
$V_{w}\times cos\left(O_{w}\right)$	Preprocessing function for wind speed

**Table 2:** The interacting data between X-Plane and the reinforcement learning (RL) program.

Data Received From X-Plane	Data Sent to X-Plane
Longitude	Operations on elevator
Latitude	Operations on aileron
Altitude	Operations on rudder
Angle of pitch	Operations on throttle
Angle of roll	
Heading	
Velocity along the longitude	
Velocity along the latitude	
Velocity along the altitude	
Angular rate of change of pitch	
Angular rate of change of roll	
Angular rate of change of heading	
True air speed	

**Table 3:** The detailed configuration of the proposed RL algorithm.

Memory capacity (one of the 24 h)	20,000
Learning rate (actor)	$9^{-4}$
Learning rate (critic)	$1^{-3}$
Optimization method	Adam
Discount factor γ	0.9
The actor replacement interval $I_{a}$	800
The critic replacement interval $I_{c}$	600
Soft update factor τ	0.98
Batch size	32

## Data Availability

Not applicable.
